# Sustainability of Digital Home Care and Health Care Services in 2 Case Studies in Finland: Combined Climate and Social Impact Assessment

**DOI:** 10.2196/71379

**Published:** 2025-10-08

**Authors:** Helinä Melkas, Jáchym Judl, Janne Pesu, Satu Pekkarinen, Riika Saurio

**Affiliations:** 1 Department of Industrial Engineering and Management, Lappeenranta-Lahti University of Technology, Mukkulankatu 19, Lahti, 15210, Finland, 358 405881400; 2 Finnish Environment Institute, Helsinki, Finland

**Keywords:** digitalization, climate impacts, social impacts, health care, home care, quantitative assessment, qualitative assessment

## Abstract

**Background:**

Digitalization is seen as a way to reduce the negative environmental impacts of health care production, but research is still limited.

**Objective:**

This study focuses on the assessment of the sustainability aspects of digital services in home care and health care. It demonstrates the approach to identify the climate impacts and social impacts—both positive and negative—on a selection of digital home care and health care services, such as medicine robot services for older home care clients, through 2 Finnish case studies.

**Methods:**

Impacts are identified from interviews and statistical data collected from public service providers and technology suppliers using both quantitative and qualitative assessments.

**Results:**

While a well-planned and well-implemented digital service is likely to be a climate-friendly option, every digitalization action carries at least some negative impacts. The design, architecture, and practical implementation of these services greatly affect their climate and social impacts.

**Conclusions:**

This study uses a novel combination of impact assessment methods, highlighting the importance of qualitative understanding alongside quantitative approaches for interpreting results, especially when numerical data are limited. Advocating for multimethod impact assessments is crucial to properly capturing the service context and promoting holistic sustainability thinking.

## Introduction

### Background

Digitalization of health care and care services typically aims to improve the availability of services for citizens in need. It is also viewed as a way to increase sustainability. Digital health care and care services, such as remote monitoring, medicine robots, automatic reminders, digital appointments, and other electronic services, as well as management of one’s own data, have been found to support the prevention of health problems, self-assessment of service needs, and independent living [1,2]. An increasingly prevalent aim of digitalization strategies is for services to be implemented in a socially, environmentally, and economically sustainable manner (eg, [3]). The digitalization of health care and care services faces numerous challenges, including diverse ethical issues, data protection and privacy, and the fact that these services are typically related to citizens’ challenging or fragile life situations [4]. Despite these challenges, the enabling role of digitalization in public healthcare and care services is intriguing due to its broad scope, societal significance, and potential to reduce the need for human labor (eg, [5]).

This study focuses on the climate impacts of the digitalization of public health care and home care services and its potential to mitigate the negative impacts of current practices, with an emphasis also on qualitative social impacts. Digitalization is seen as a way to reduce the negative environmental impacts of health care production (eg, [6,7]); however, research is limited, and assessment methods still require development, as discerning the impacts requires a multidisciplinary and holistic approach in both research and practice. It is unclear whether, for example, the increased electricity and rare material use due to digitalization will be compensated for by efficiency gains and sustainable behaviors fostered by digital innovations. Moreover, digitalization may, for instance, consolidate incumbents’ power and unsustainable practices [8]. There is a tendency to consider digitalization as something automatically positive from an environmental perspective, which further highlights the need for robust and user-friendly assessment methods (eg, [9], focusing on evaluation).

In addition, the uptake of new services in health care requires a human-oriented perspective that takes into account patients or clients, their informal caregivers, and professional caregivers [9]. User adjustment is crucial when implementing disruptive technologies and practices, as their introduction entails more than mere adoption: users need to integrate novelties into their practices, routines, and organizations [10]. A detailed consideration of both the positive and negative impacts of digitalization still requires well-informed methods that include a strong human perspective.

Our main focus in this study is on the assessment of the sustainability aspects of digital services in home care and health care. Through 2 Finnish case studies, this study demonstrates the approach to identify the climate impacts and social impacts—both positive and negative—on a selection of digital home care and health care services, such as medicine robot services for older home care clients. The examination of other technologies and services, such as remote health care appointments, plays a supportive role. Digitalization plays an increasingly central role in the development of home care and health care services; however, its implementation should be supported by concrete tools to assess its impacts. Based on empirical data, the results of both quantitative and qualitative assessments are presented. The latter also includes social impacts related to and intertwined with climate impacts.

### Sustainability of Health Care and Care Services

Until recently, the sustainability of health care and care services has been regarded particularly from the point of view of economic and social sustainability. However, the integration of environmental sustainability is inevitable [11] to recognize the connections between the climate crisis and the challenges of health care and care. Health and well-being, on the one hand, and environmental sustainability, on the other, are still often examined separately, although they have many mutual effects. The health care sector is directly affected by climate change impacts on patients and, at the same time, is responsible for over 4% of greenhouse gas emissions globally [12, 13].

Finland’s journey in health care and care service digitalization has spanned decades, beginning in the 1960s, and gradually expanding to support client and patient care [14]. A major turning point came around the year 2000 with the introduction of a national strategy to advance digital technologies, and over the past 2 decades, digital solutions have become an essential part of health care and care delivery. Digitalization is driven by a combination of historical development, national strategies and reforms, technological advancements, efficiency and accessibility improvements, and support for the rapidly aging population (eg, [14]). A comprehensive national reform of health care and care services, social services, and rescue services of 2023 marked a significant shift also in the trajectory of digitalization. Digital tools are widely used in both primary and specialized outpatient care across the public and private sectors [15], but the environmental sustainability of digital health care and care services is still quite an emerging perspective.

While interest in “sustainable health care” has grown globally, there is still no consensus on its definition [11]. The definition (scope) of health care can also vary across countries and their different service systems. This paper concerns older people’s home care as well as health care services, and in most instances, “health care” and “care” are thus used side-by-side in this text. The sustainability transition in health care and care systems is systemically linked to the transition in related systems on their periphery, such as the living environment, traffic, construction, education, and food production [13,16]. However, the core business of health care and care-related persistent problems, systems, and structures remain largely unchanged [16]. A transition perspective may help identify more promising ways of understanding and addressing the sustainability issues faced by contemporary health care and care systems [17].

Knowledge is needed about what constitutes sustainable digital health care and care services and how they are defined (eg, [5]). There are both challenges and opportunities in how digital technologies are integrated into existing health care and care services and systems, including the initiation of new services [18]. The structures and support systems of health care and care play a central role here; the sustainability of digital innovations depends on whether their integration is conducted in a way that supports their long-term stability [6,9]. Contextual factors, such as financing, organizational support, and people’s capabilities, capacity, and education, affect the integration process [19,20].

### The Broad Field of Digitalization

When considering environmental sustainability, it is essential to consider the digitalization of health care and care services from a broad perspective [7]. There are diverse types of services and patient or client groups, technologies and systems, and programs and infrastructures, and services are offered and used in very different circumstances and environments, from homes to various institutional settings. Nordic Innovation [21] reminds us that in addition to sustainable service environments and sustainable technologies, sustainable health care covers sustainable behavior and practices. From a human perspective, sustainable health care is related to sustainable health (well-being and disease prevention) and environmental health (health risks both outdoors and indoors and how they affect an individual), and it refers to treatments that take place after an individual has fallen ill (both out- and in-patient care), according to Nordic Innovation [21].

On the other hand, the digitalization of health care and care-related processes and improved cooperation (eg, between hospitals and product suppliers) can support decreasing the carbon footprint. Improved data availability may render decision-making in hospitals and healthcare centers more efficient. Beyond building design, it is crucial to consider the sustainability of materials used in new technologies, which necessitates procurement expertise. In addition to the above-mentioned perspectives outlined by Nordic Innovation [21], sustainability should not be limited to contexts in which an individual has already fallen ill and become a care receiver. If preventive services and technologies are taken more broadly into consideration, it should be possible to better harness the positive potential of digitalization and thus achieve more systemic results [10]. This could involve, for example, the earlier introduction of proactive digital services in home environments [22].

Digitalization in health care and care is also characterized by the so-called emerging technologies that are currently used to some extent in these services. The Finnish Ministry of Transport and Communications has determined that by means of new solutions implemented through technologies such as robotics, artificial intelligence, and automated systems, along with their support technologies (eg, cloud services), negative environmental impacts in different sectors (including health care and care) could be decreased [23]. Opportunities for these technologies have been recognized in health care and care services, but the use of robots, for example, remains relatively rare [24]. Ethical and legal issues, among others, may cause challenges in this area [25]. More traditional technologies can also be used to implement remote health care and care (also called eHealth, digital health, telemedicine, telehealth, or distance-spanning solutions), potentially affecting people’s mobility and use of buildings. Indeed, remote health care and care services of different types are being increasingly adopted in various countries. A significant increase in their supply and use resulted from the COVID-19 pandemic [26]. Our impact assessments covered both emerging and more traditional technologies, while this paper concentrates on the emerging technology of medicine robots.

## Methods

### Background to Case Studies

This empirical study focuses on cases from the 2 Finnish regions of Päijät-Häme and South Karelia, particularly on home care services for older people (medicine robot and video call services), as well as remote health care appointments in dental care, nutritional care, and mental health and substance abuse services for children and youth. Most of this paper concentrates on the study of medicine robot services due to their enormous potential for global adoption, as well as their innovative use of remotely controlled hardware to complement or even substitute for human labor. Both cases are introduced to provide sufficient context for our work. Päijät-Häme is in Southern Finland, while South Karelia is in Southeast Finland. Both regions are rather sparsely populated, with long distances to services.

### Case 1: Digital Home Care Services, Especially Medicine Robot Services

In Case 1, the home care services are provided by the older adult care services and rehabilitation unit of the Päijät-Häme Joint Authority for Health and Well-being (currently Well-being Services County of Päijät-Häme). The Päijät-Häme Joint Authority for Health and Well-being provides services for the more than 200,000 residents of the region, with its 7000 employees. Part of the home care services in this region is produced as remote care services. The remote care and technology unit, Severi, serves regular home care clients with the help of video calls and medicine robot services. Severi employs both nurses and assistant nurses. The Päijät-Häme region is a pioneer in remote care arrangements in Finland (for further information on the regional home care ecosystem, refer to [27]). At the time of data collection (2022), there were 257 medicine robot clients (15.3% of all clients) and 173 video call clients (10.1% of all home care clients). Medicine robots and video call services are not quite new in the field, although their use has proliferated quite rapidly. The environmental sustainability or climate impacts of medicine robots had not been studied in the region.

The primary use of medicine robots is as medicine dispensers; however, they can also be used to provide various reminders. Medicine robots are available to home care clients free of charge and can help provide regular medications just on time. A home care professional generally fills the robots for a period of 1‐2 weeks. In the event of possible disturbances, such as power outages, the device sends an alert to a care professional. The devices also trigger an alarm if a client does not take the medicine offered or if there is an attempt to break into the device. The medicine robot can function as a service on its own or complement other home care services. The medicine robot technologies in use in the region are Evondos (since 2016; [28]) and Axitare (since 2020; [29]). The medicine robot requires mains power but no fixed internet connection, thanks to a built-in mobile modem. Employees can access the backend, as well as the robot itself, using a tablet, computer, or mobile phone with an internet connection, which the service organization must acquire, along with purchasing the overall service and a maintenance contract with a technology provider. One of the medicine robots dispenses medicine bags, while the other uses dose cups. The purpose of the medicine robot service is to replace one or more visits by care professionals, but not necessarily all.

Video call services are conducted on a 10-inch tablet provided to the home care client. The service provider conducts a needs assessment and, based on the service criteria and the assessment, tailors the client’s services. This type of service can be offered to a client whose functional ability enables them to act according to instructions. A care professional contacts the client at a previously agreed time. Video call services can be used to guide and ensure medication intake (the client’s functional ability permitting) and to monitor nutrition. The clients can also participate in group events organized by the service provider, such as rehabilitation exercise sessions. The tablet only has the video call app installed; it cannot be used for anything else. The care professionals use computers with separate displays and headphones when calling clients. An ordinary call lasts about 10 minutes, although 15‐20 minutes is also possible (with group activities lasting 30 minutes). Video call services can be combined with other services, such as a safety alarm wristband, but such possible services were not taken into account in this study’s calculation, nor were speakers, which can be provided to clients who are hard of hearing.

### Case 2: Remote Health Care Appointments

The South Karelia Social and Health Care District (currently Well-being Services County of South Karelia) provides several types of remote health care appointments. Our assessments covered the services of three units: (1) dental care (dental examinations of 1-year-olds), (2) nutritional care, and (3) mental health and substance abuse services for children and youth (family counseling and youth support). The South Karelia Social and Health Care District produces public social and health care services, employing about 5000 staff members to serve approximately 129,000 residents of the 9 municipalities in the South Karelia region. The 3 units represent both centralized and decentralized services. Remote health care appointments are available to all residents as an alternative to traditional on-site client or patient visits. The care professional offers the possibility of a remote appointment that is conducted with the help of either VideoVisit or Microsoft Teams. VideoVisit (currently Oiva Health, [30]) is a digital care platform used in the Nordic countries; it is designed for primary health care, social care, hospital health care, and long-term care services. The client’s significant others or designated support persons can also participate in remote appointments. If needed, professionals from several different services can attend as a network (especially in mental health and substance abuse services). At the time of data collection, the percentages of remote appointments were as follows: (1) dental care (1-year-olds): 0.2% of all contacts (a 1-time visit for this age group), (2) nutritional care: 13.1% of all contacts, and (3) family counseling and support for youth: 11.3% of all contacts.

The original aim of the remote appointments was to improve the availability of services in a region where travel distances can be long; however, the COVID-19 pandemic accelerated adoption. No special dedicated equipment is required to provide or access the service, only a regular computer, a smartphone, or a tablet connected to the internet.

### Research Methods

Data were collected for the qualitative assessment, the development of a calculation model, and the establishment of a quantitative assessment method. The collected data consisted of interviews with service providers and technology suppliers, as well as documents provided by the case organizations. These documents included log information about the use of digital services, evaluation reports, planning documents, and statistics and annual reports related to service use and development in the region. A list of relevant knowledge needs was drafted to guide the data collection, aiming to gather comprehensive information on both direct and indirect impacts. From the perspective of clients, care professionals, and the service system, data collection focused on a detailed description of the service (eg, devices used, architecture of the backend system, daily organizing of the service, and work environments or spaces), as well as the reasons for the introduction or digitalization of the service (economic reasons and those related to service quality) and its impacts (both intended and other impacts in a broad sense). Information was also collected on how the services had been organized in the past, how the digitalization of the service changed the operations of the affected parties, and patient or client satisfaction.

From the perspective of companies (technology suppliers), data collection addressed a description of the service from the company’s perspective and matters related to manufacturing materials, production, electricity consumption, recycling, lifecycle of materials or parts, backend services, and network use. Similar knowledge needs applied to Cases 1 and 2, but the details were case-specific. The exact content of the interviews was tailored according to each interviewee’s role and service type.

The interviews were conducted in January-March 2022 as remote Teams interviews. Most of the interviews were conducted individually, although a few were carried out as pair interviews. The interviewees were selected to obtain the most comprehensive information about the climate and social impacts from perspectives relevant to the services in question, such as backend and frontend services. The interviewees represented a wide range of professions and several organizational levels, including management, development, and planning of services, supervisors of patient or client service work, employees working in patient or client services, technology trainers, and technology suppliers.

The interviewees were selected in collaboration with the 2 service providers’ contact persons so that their perspectives were complementary and covered the whole of the services in a sufficient manner. In Case 1, there were 12 interviewees: 9 from the service provider (assistant nurses, a registered nurse, an immediate supervisor, and management representatives) and 3 from the technology suppliers. In Case 2, there were also 12 interviewees: 11 from the service provider (a service designer, developer, management representatives, immediate supervisors, and personnel from the 3 units studied) and 1 from the technology supplier.

The interviews were recorded and transcribed. Due to different client or patient groups and service types, Cases 1 and 2 together offer a multidimensional picture of the digitalization of public health care and care services. While most of the results concern the medicine robot services, both cases are discussed.

### Quantitative Assessment of Climate Impacts

The quantitative assessment of climate impacts was based on life-cycle assessment (LCA) methodology. LCA is a well-established method for studying complex value chains in order to understand potential environmental impacts. In LCA, all relevant elementary flows entering or leaving the studied system are characterized according to a defined impact category. In this assessment, the focus was exclusively on the impact category of climate impacts, using the indicator Global Warming Potential (*kg CO_2_e)*. LCA enables quantification of the potential environmental impacts of the entire product system, including upstream impacts. This is crucial when studying systems that are global in nature.

It was not possible to assess and compare complete services before and after digitalization due to limitations of data and the incremental way in which the studied services had been digitalized. It was therefore decided to calculate the impacts of the current digitalized services and then compare those impacts to commonly understood and case-relevant climate impacts of traveling via car or bicycle.

In LCA, impacts are calculated per defined functional unit, which is a measure of the performance of the product system being studied. It provides a reference to which all inputs and outputs of the system can be related. The functional unit was defined as 1 year of service usage of 1 client or patient in continuous services and a single session in other services. This definition of the functional units also facilitated the comparison of the climate impacts of digital services to the climate impacts of potentially saved travel.

**Figure 1.**
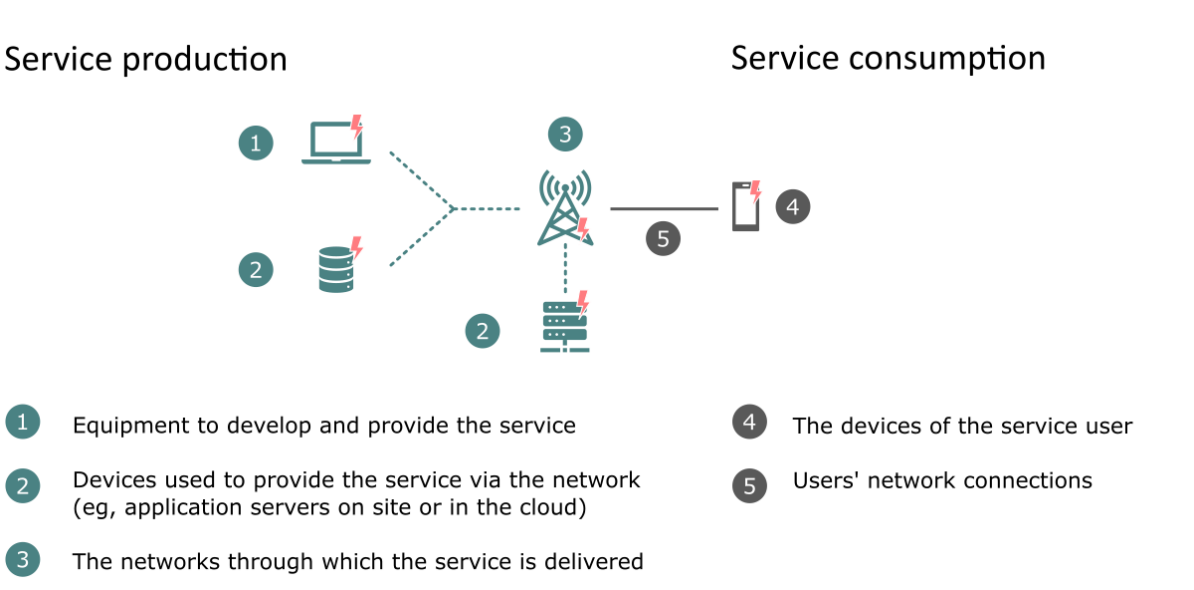
Framework for the assessment of climate impacts of digital services.

Our framework for the quantitative assessment of the climate impacts of digital services is shown in Figure 1. This is a highly simplified framework; however, it still adequately describes all the digital components of the service. The production of a digital service requires user IT equipment, an internet connection, and servers. The consumption of a digital service requires an internet connection and an access device. The access device can either be dedicated to the service (eg, a medicine robot or dedicated tablet) or not (a personal computer, smartphone, or personal tablet used for other purposes as well).

This simplified framework allowed for the assessment of all the studied services based on the collected data. Calculations based on detailed solution architecture would have been practically impossible because, for example, the network component of a service can include hundreds of connections and processing units.

### Qualitative Assessment

For the qualitative assessment, the interviews were analyzed using content analysis to identify climate impacts and related social impacts separately. The qualitative assessment method used in this study is based on the principles of human impact assessment (HuIA; eg, [10,31]). The assessment of human impacts can be used to structure new perspectives and to describe solution options. HuIA takes into account the fact that impacts can be planned or unintended and can be formed as a result of long chains or networks of impacts [31]. In this study, assessing impacts on people implies, for example, examining chains of impacts of digitalization, such as connections to well-being, relationships between people, and work. HuIA is comprehensive in nature—impacts are not limited in advance, and efforts are made to comprehensively identify them and make them visible. This approach has been used in studies on the digitalization of older adult care and health care services concerning traditional technology, such as safety alarm systems [32,33] and emerging technologies, such as care robots [10]. Its essence is to identify positive, negative, and neutral impacts on different groups of people involved holistically.

**Figure 2.**
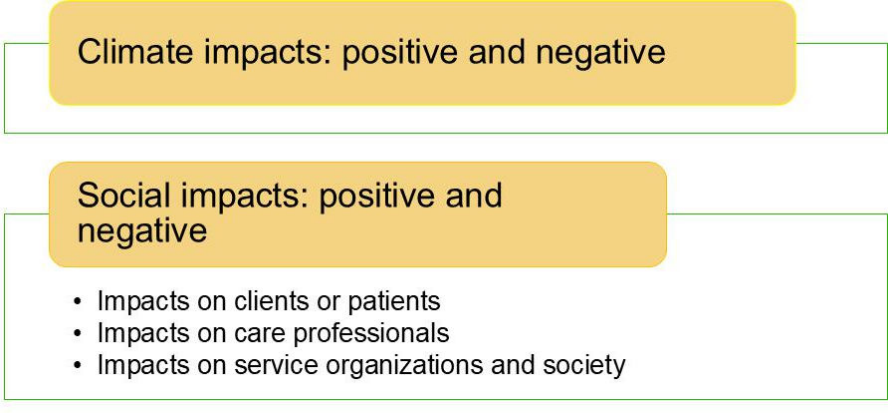
Categorization of the qualitative results.

An inductive thematic content analysis [34] of the data was conducted. Accordingly, the transcribed text and notes were thoroughly read to capture the features associated with the research topic. The sentences and paragraphs from the interviews that were assessed as interesting or meaningful regarding the phenomenon under study were identified and marked, and initial codes were generated across the dataset. In this study, the codes were grouped into positive and negative climate impacts and related social impacts. The positive and negative social impacts were further grouped into impacts on clients or patients, impacts on care professionals, and impacts on service organizations and society (Figure 2). These were checked against the codes and the entire dataset for relevance.

### Combining the Assessments

The interviews and the related document collection provided information on the context, components, and whole of the services, including the service production and service consumption, from different perspectives. They also enabled the development of a simplified framework for the quantitative assessment and the definition of the functional units for it.

Based on the interviews, statistics, and other documents, the material and energy inputs required by both the information and communication technology system and the physical service were investigated. For the calculation of climate impacts, quantitative data on direct and indirect factors were collected as comprehensively as possible. Comparable data about the service and deliverables before digitalization, in particular, proved to be difficult to obtain from the case studies. The calculation was made case by case on the basis of the data obtained.

The interview material provided a rich basis for the qualitative assessment, including analysis and results on how people’s actions and ways of doing things affected the impacts. The quantitative and qualitative assessments fed into each other, at different phases, in gaining a comprehensive view of the topic of the study and understanding how mixed methods can be used and why they should be used in studies of digital services. As a practical outcome, the combination of the quantitative and qualitative approaches also led to the development of an online calculation service for practitioners [35-37].

### Ethical Considerations

Ethical standards were maintained throughout the research. All interviewees provided informed consent for participation and subsequent interviews, and leaving midsession was permitted. Research permits were obtained from the appropriate authorities, and participants’ privacy and confidentiality were maintained. The study was approved by the Ethics Committees of the Päijät-Häme Joint Authority for Health and Well-being (approval no D/38/13.00.00.00/2022) and the South Karelia Social and Health Care District (approval no EKS/583/13.01.05/2022).

## Results

### Quantitative Assessment Results Regarding Climate Impacts

#### Medicine Robot Services

**Figure 3.**
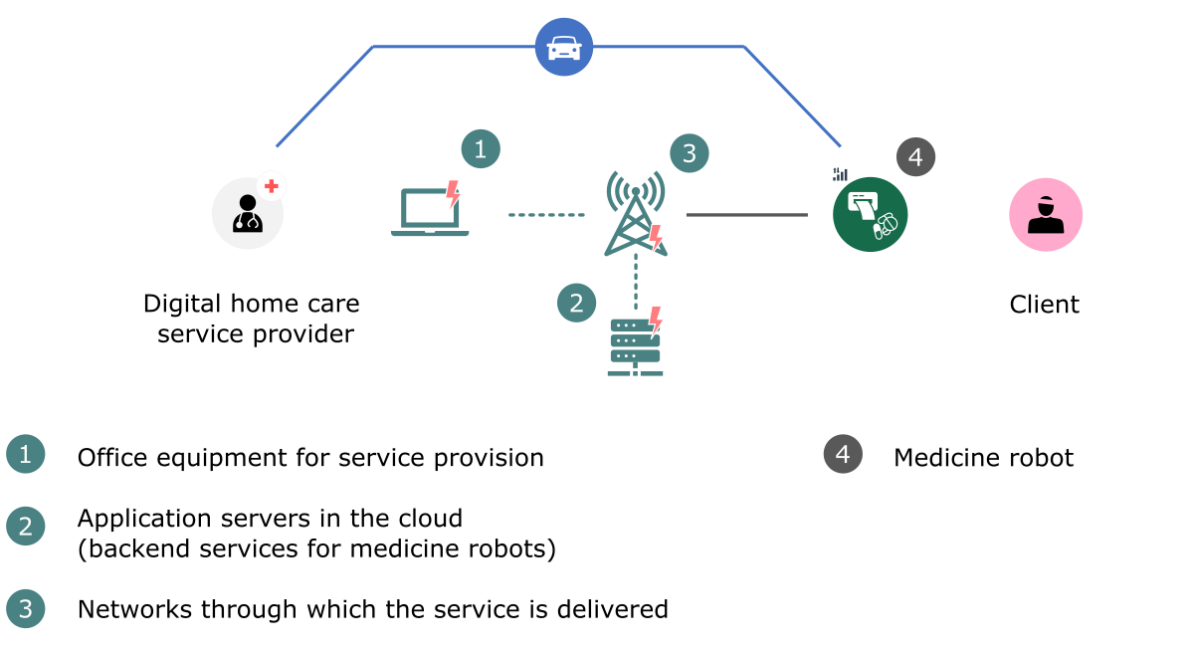
Medicine robot service components for impact assessment.

The climate impacts of medicine robot services were calculated for a functional unit of 1 year of use of a medicine robot by a client who takes medication 3 times a day. Figure 3 shows the basic components of the service. These are the same components as in the framework shown in Figure 1, with the addition of physical visits to refill the medicine robot.

**Figure 4.**
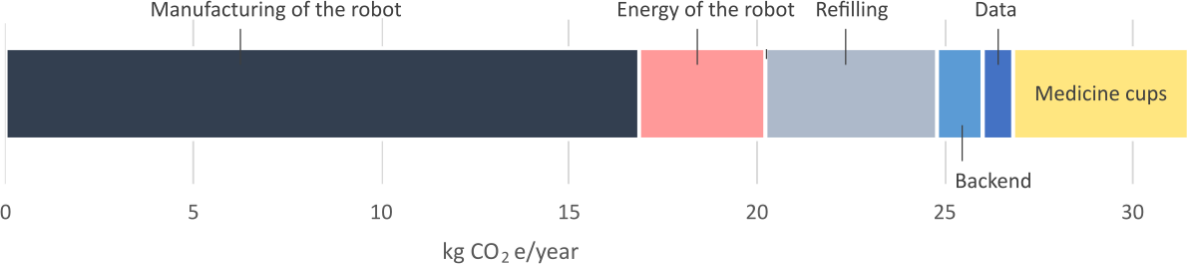
Climate impacts of a medicine robot (aggregated). CO_2_e: carbon dioxide equivalent.

The climate impacts were calculated for both medicine robot types, and the overall results were quite similar. The aggregated results are shown in Figure 4. The manufacturing of the robot dominates the climate impacts. Refilling and energy use together account for less than half compared with the impacts of manufacturing. The backend and the mobile data represent only a small contribution to the overall climate impacts. Medicine cups—used in one of the robots—cause more impacts than the energy consumed by the robot. The overall carbon footprint of a yearly medicine robot service was estimated to be about 30 kg CO_2_e (carbon dioxide equivalent).

**Figure 5.**
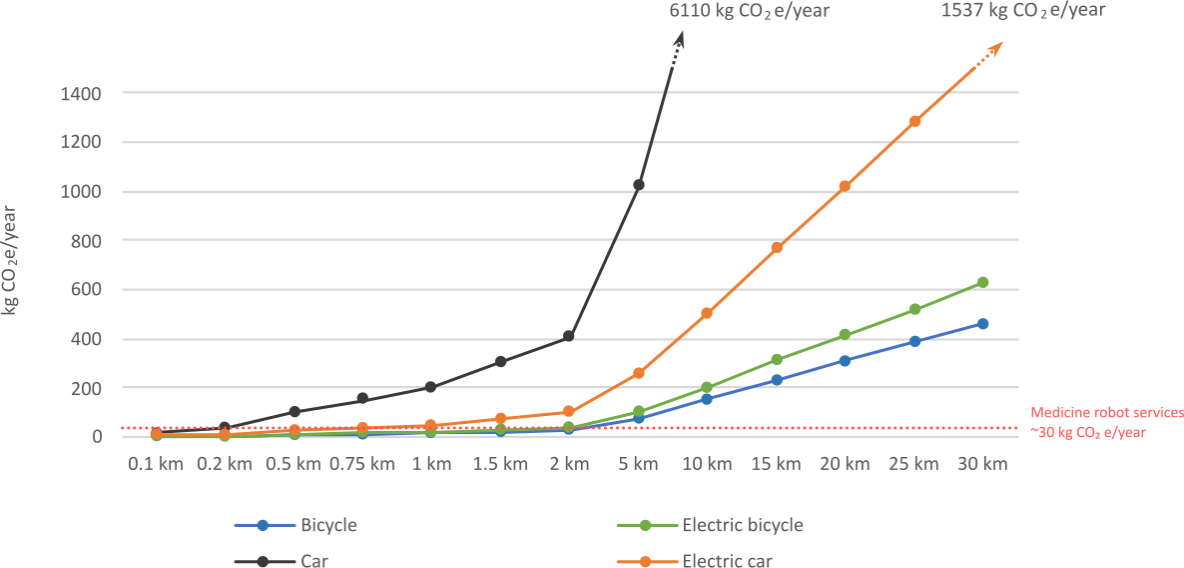
Comparing the climate impacts of the medicine robot service with the climate impacts of avoided travel. CO_2_e: carbon dioxide equivalent.

The medicine robot reduces the need to travel. Clients without a robot typically need 2 visits per day, while the robot requires a refill visit every 2 weeks. Figure 5 compares the climate impacts caused by the medicine robot service to alternative means of transport to fulfill daily medication needs.

In short, the medicine robot is a climate-friendly option when the distance to the client by car (even electric) is more than a kilometer (2 kilometers by bicycle).

To conclude, the medicine robot demonstrates good potential from the perspective of reducing climate impacts. It is especially beneficial for rural areas, where client visits necessitate car usage.

Providers of technology and services are key to reducing carbon footprints. Manufacturing of the medicine robot causes over half of the assessed climate impacts. The carbon footprint of manufacturing depends not only on the design and use of materials, but also on its lifespan. Operational energy and data usage of the robot should also be optimized, but they have less impact on the carbon footprint. Requiring carbon footprint calculations from technology and service providers would be an effective way to ensure overall green services.

#### Other Services

**Figure 6.**
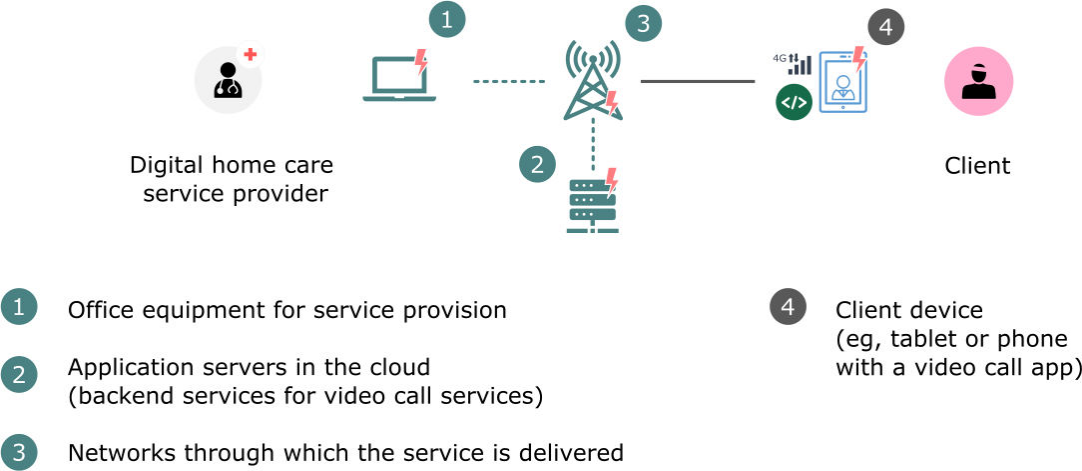
Video call service components for the impact assessment.

The other services assessed were video call services, consisting of the same components as in the framework (Figure 1). The basic components of video call services are shown in Figure 6.

Remote health care appointments occur infrequently; therefore, a functional unit of an appointment was selected. This approach allowed for a direct comparison to traveling to an appointment at the health care facilities. Clients use their own smartphones, tablets, or computers for video appointments; therefore, the carbon footprint share of client device manufacturing is negligible. The calculated carbon footprint of a remote health care appointment was 0.044 kg CO_2_e, which is about the same as driving 1 kilometer with an electric car. Remote health care appointments have a carbon footprint, but a low one compared to driving to an appointment.

Video call services for home care clients have the same basic components (Figure 6); however, calls occur frequently, and clients use a dedicated tablet reserved for this use only. The assessment was carried out for a functional unit of annual use of the video call services. The calculated footprint was 61 kg CO_2_e, which is about twice that of the medicine robot services. The comparatively high climate impact is mainly due to the dedicated tablet with a short lifespan on the client side. The manufacturing of the tablet causes 73% of the yearly footprint. Video call services with a dedicated tablet are a good option in rural areas where distances to clients are longer; however, the results also show that a digital service is not automatically climate-friendly.

### Qualitative Assessment Results

#### Positive Climate Impacts

##### Medicine Robot Services

Medicine robot services enable reducing the kilometers driven by care professionals. The number of care professionals’ home visits may decrease from as many as 60 visits per month per client (to administer morning and evening medicine) to 2 visits (to refill the robot). In the studied regions, the distance from the home care office to the clients’ homes can be tens of kilometers. A care professional noted:

I personally find these [medicine robots] useful. These have been well received in the work community. Suitable clients are suggested from the field. It’s frustrating to drive 50 minutes to give the morning medicine.

Usually, only some of the visits are replaced. Medicine robot services provide the opportunity to save on care professionals’ protective equipment (eg, disinfectants, gloves, and masks). In addition, the medicine robot service may decrease medicine wastage. When only the right amount of medicine is dosed, there is no need for large medicine packages that may expire if the medicine is no longer needed. One aim is also to optimize routes and visits to clients’ homes. The life cycle of medicine robots is quite long (7‐8 years). The devices are considered durable and are repaired by replacing worn parts. The devices are reused by other clients, and the components are recycled.

##### Other Services

Video call services (including both home care services in Case 1 and remote health care appointments in Case 2) also enable reducing the total kilometers driven, either by the care professionals (Cases 1 and 2) or the patients (Case 2). The video call services in Case 1 can decrease the number of home visits (eg, measuring and dosing visits related to older adult care). If the client also has a safety alarm system, the video call service can be used to check the client’s situation when an alarm has been triggered before driving to the client’s home. Just as in Case 1, in Case 2, the service provider organization operates in a large geographical region, so remote appointments reduce the traveling of both care professionals and clients. This impact is mainly related to clients’ traveling because the appointments in question have traditionally taken place in health care centers, not in clients’ homes. According to the interviewees, remote appointments are popular in the region, especially in rural areas.

In both Cases 1 and 2, the services do not require care professionals to use care equipment or protective equipment (eg, disinfectants, gloves, and masks); thus, the amount of waste is lower. According to the interviewees, actual savings on space have not yet been achieved, but there is more flexibility in the use of the workspace; for example, an ordinary office is sufficient for a remote dental care appointment. In Case 2, space savings were noted to be likely in the future. Paper use has decreased somewhat, as written materials are mainly delivered in electronic format. However, clients may print out the materials themselves. Not all materials exist in electronic format. Before the remote appointments, all materials were mailed to clients. As with the medicine robot services, one aim of the video call services in Case 1 is to optimize the routes and visits to the clients’ homes. The life cycle of the video call tablets is about 3 years. The materials are recycled, and this service reuses the devices for other clients.

### Negative Climate Impacts

#### Medicine Robot Services

The negative climate impacts of medicine robot services are related to both energy consumption and the manufacturing of the necessary service components (Figure 3). For the medicine robot, a battery is needed in addition to mains power to ensure an electricity supply during power outages.

When the device is brought to a client, guidance is always given by a care professional. If needed, guidance is given several times in the beginning. The interviewees noted that unnecessary driving sometimes occurs, which could be avoided with better planning. Ensuring that care professionals possess the necessary technical expertise is vital so that the professional who is present in the client’s home can help when problems arise without the need to call for assistance. This is also vital from a more general perspective, as a technology supplier noted:

Thorough training and support produce [positive] environmental impacts by ensuring that the device is not left unused or that there is no return to giving the medicine on site.

Even though the medicine robot generally reduces driving, there are times when error messages or alarms require a home visit. Most of these situations can be checked by phone, such as when the device triggers an alarm due to medicine not being taken. It is rare to have to call for a technician to repair the device. Medicine robot services do not affect the number of workspaces, as the services were provided earlier at clients’ homes.

#### Other Services

The negative climate impacts of video call services (again, including both home care services in Case 1 and remote health care appointments in Case 2) are also related to both energy consumption and the manufacturing of needed service components (Figure 6). As for the tablets used in home care in Case 1, when the device is brought to the client, guidance is always provided by a care professional, several times if needed. Again, good planning is the key to avoiding unnecessary driving.

Despite the decrease in driving, negative climate impacts nevertheless arise. Different types of support services are necessary for remote health care appointments, which increase negative impacts. The number of workspaces is not directly affected, either because the services have been provided in the clients’ homes (Case 1) or because some space is needed for remote appointments as well (Case 2). The interviewees, however, assessed that the need for workspaces may decrease in the future.

### Social Impacts

Health care and home care services are a challenging field in which to conduct impact assessments, and the importance of including social impacts was reinforced by the fact that diverse, intertwined, and multidirectional impacts became visible in the study. Social impacts for Cases 1 and 2 are reported together and categorized into positive and negative impacts at different levels—clients, employees, organizations, and society (Tables1  2). Such impacts may be intended (in line with the aim of the digitalization of actions) or unintended. A few descriptive quotations from the interviews are included as examples.

**Table 1.** Positive social impacts (Cases 1 and 2).

Category	Positive social impacts per case
Clients or patients	Case 1: savings on service fees, improved accuracy of medication and care visit timing, decrease in medication errors, maintaining a sense of independence and autonomy, opportunity to focus on social interaction during video calls, and opportunity to participate in various activities from home. Case 2: regional equality, accessibility of services regardless of place of residence, flexibility, and saving time, money, and effort spent on traveling.
Employees	Both cases: reduction in the time required for traveling and mechanical tasks, easier planning of time use (more time for actual nursing work rather than, eg, changing protective equipment and disinfecting between client visits). Case 2: networked multiprofessional collaboration is more flexible remotely; remote work was felt to increase workplace well-being.
Service organizations and society	Both cases: more rational allocation of resources (face-to-face visits to those who really need them), possible increase in the appreciation of care work and its attractiveness. Case 2: reduction of canceled appointments and no-shows. Practical experience has revealed new opportunities for remote appointments. Remote appointments and remote work are considered assets for recruiting.

**Table 2.** Negative social impacts (Cases 1 and 2).

Category	Negative social impacts per case
Clients or patients	Case 1: not suitable for everyone (eg, possible delusions); perceived unsuitability of the devices (size and appearance) to the home environment. Case 2: inequality of citizens (digital skills, opportunities to acquire devices, and internet connections). Remote appointments can exacerbate or sustain problems (eg, fear of social situations may not be visible).
Employees	Both cases: change of work, new tasks (eg, responsibility for technology and assessment of service needs). Change requires learning, which can be overwhelming. Case 1: no decrease in total workload because the number of clients is increasing all the time; easy visits have decreased; challenging ones have remained. Case 2: it is not possible to observe the whole person or the living conditions when working remotely. Possible deterioration in interaction.
Service organizations and the society	Both cases: challenges related to work culture and bringing technology into service processes (increased complexity of management); new and old ways of working collide (eg, engaging and assisting personnel, procurement expertise, support services, data security, data ownership and storage, and integration of different technologies).

Positive social impacts included the preservation of clients’ activity and independence, regional equality regardless of place of residence, the rationalization of employees’ work, increased work flexibility due to a decrease in commuting time, and a more reasonable allocation of society’s resources. A care professional noted:

Medication errors have decreased even though double checks are in place. Some clients have gained independence, meaning they can live at home on their own. Lots of things. Lonely clients may have made friends. And I think a couple of relationships have even blossomed.

Negative social impacts included issues related to client inequality (such as differences in digital skills, financial opportunities, or the service being unsuitable for certain clients), increased employee workload during the transition to new working methods, and increased management challenges and complexity.

The detailed social impacts depend on the type of service and technology (refer to Tables1  2). Medicine robot services, for example, such as the use of home care technologies in general, have led to savings in clients’ care fees. Clients found the devices easy to use, as they are largely automatic, reliable, and require no technical skills. The use of the medicine robot also led to other positive outcomes, such as improved quality of medication care and fewer medication errors. The medicine robot always administers the medicine at exactly the specified time, which improves the accuracy of medication. When a care worker administers the medicine, the timing may vary. The robot also relieves the client of the mental pressure of remembering to take the medicine. Several reminders can be given by the robot. Furthermore, the service provides a sense of participation and accomplishment. Some clients do not like visits by care workers, and the medicine robot enables clients to maintain their independence while ensuring access to care.

The perceived unsuitability of the devices to the home environment was mentioned as a negative social impact from the clients’ point of view. The large size of the medicine robot may come as a surprise, and clients may perhaps consider it ugly and inappropriate for their interiors. The medicine robot also does not promote social interaction, unlike a home care worker’s visit or a video call service. Often, the medicine robot and the video call services work in tandem for the client. It is important to consider for whom the technology is suitable, especially in the case of people with memory and mental illnesses, as they may have delusions about, for example, spreading the image of a video call to outsiders. The client may also unplug the medicine robot or turn it upside down, causing the medicines to get mixed. Both situations trigger alarms. Technical problems, for example, when establishing a video call connection, also appear as negative impacts, although they are typically caused by network overload or a bad audio line. However, such problems are relatively rare.

The first reaction of clients and their loved ones to the introduction of technology to home care (Case 1) is often negative, as the care may be thought to be worse when provided with the help of technology. A care professional noted:

Clients and their families have individual attitudes. Often, there is initial resistance, but once they try it and learn how it works, they realize how convenient it is, and their attitude usually becomes positive, and they start using the service.

The implementation of the technology in Case 1 always starts with a 2-week trial period, after which it is possible to stop using it. However, most clients were satisfied after the trial period and wanted to continue using the device. In Case 2, the negative social impacts differ somewhat; remote health care appointments can exacerbate or sustain clients’ problems, and it is not possible to observe the client’s situation as a whole. Interaction may deteriorate as well. A technology supplier explained management challenges:

The transition from traditional models to the use of technology is a change that requires change management and organizational involvement. […] It is often underestimated that once the purchase has been made, home care staff are told to start using the robots, and then there are many fears about how this will affect their work. “What if I don’t know how to use this service?” Political decision-making, courage, change management, and organizational involvement are needed. […] Not just “Start using the robots.”

## Discussion

### Principal Findings

The quantitative assessment showed that a well-planned and well-implemented digital service is likely to be a climate-friendly option. Digitalization is generally considered a positive measure for the environment; however, it does not automatically lead to positive climate impacts. Every digitalization action incurs some negative impacts due to the necessary equipment and energy consumption. The design, architecture, and practical implementation of digital services greatly affect their climate and other impacts, as shown by our study.

**Figure 7.**
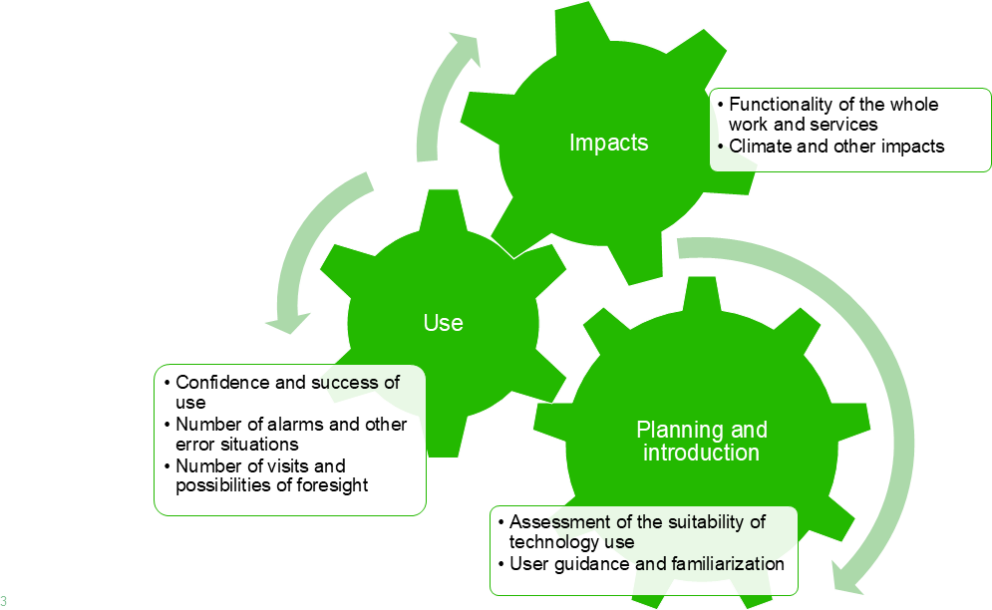
An example of the intertwined and multidirectional impacts of medicine robot services.

The qualitative assessment clearly showed the multidirectional, interconnected impacts of the studied digital services and the connections of the impacts to people’s actions and ways of doing things. This emphasizes the need for contextual understanding enabled by qualitative assessment when making impact assessments. The results showed, among other things, that it is important to adequately assess the suitability of a digital service or its underlying technology for the client or patient before implementation and to carefully familiarize all parties involved to ensure safe and successful use. This, in turn, contributes to better planning and foresight, enhancing the functionality of the services and system as a whole, which increases the potential for positive climate impacts (Figure 7).

Although this study had its limitations, as it concerned 2 Finnish case studies and a limited selection of digital services and technologies used in those, a variety of impacts were found in this study. Despite very different national service systems, services, digital technologies, etc, the findings can provide guidance and pave the way for increasingly multifaceted impact assessments and more sustainable, effective, meaningful, and balanced digital technology use in future health care and care services. Life cycle analyses and actionable strategies for a sustainable digitalization in health care systems have recently been called for by Berger et al [7] (also refer to [38] on the future potential).

In addition to Finland, health care and home care services are undergoing major changes in their operating environment in many other countries, such as increasing numbers of clients, changing organizational and service structures, challenges in finding enough staff, and financial challenges. Digitalization development is one such major change, representing a major socio-technical transition in which technological, social, and environmental aspects intertwine. Assessing the impacts of digitalization, especially before or after assessments, is therefore difficult. Even in Finland, where the health care and home care services have been digitalizing for decades, access to numerical data was also limited.

### Practical Implications

The COVID-19 pandemic accelerated the introduction and use of various types of digital health care and home care services. However, climate impacts have not been the central drivers of the digitalization of these services in Finland. According to care professionals, these issues are not yet very actively discussed. One manager noted that environmental friendliness is considered “a positive byproduct.” Wider adoption of emerging technologies (eg, robotics and artificial intelligence) will likely increase the challenges of environmental impact assessment (eg, [39]) globally. This development highlights the importance of increasingly proactive assessment activities in all countries in the future.

Based on the results, multiperspective and multimethod impact assessments should be promoted in studies of digital health care and home care services. To complement quantitative assessment methods—and aid in interpreting their results—qualitative insights into digitalization and its impacts are needed, especially when access to numerical data is limited. A systemic understanding of the service context, where “everything affects everything,” is essential to understanding and advancing sustainability thinking in the complex context of health care and home care services. Sustainable digital health care and home care services need to address social, economic, and environmental aspects. Future research should explore various types of digital health care services for diverse clients and employees across different organizations, service systems, and societies.

### Conclusion

To conclude, this study developed and used a novel combination of quantitative and qualitative methods to assess the climate impacts and related social impacts of the digitalization of health care and home care services. Digitalization is expected to play a central role in these services; however, the field is very complicated and challenging to digitalize in meaningful and effective ways. Thus, concrete practical tools are needed to assess the impacts of digitalization. The key results from 2 Finnish case studies of innovative digital health care and home care services offered a multidimensional picture of digitalization’s impacts, including both climate impacts and intertwined social impacts, on different client or patient groups and professional groups in different types of services. Crucial lessons learned for the field of health care and home care services include that well-planned and well-implemented digital services are likely to be climate-friendly options. However, every digitalization action causes at least some negative climate impacts. The design, architecture, and practical implementation of digital services significantly affect their climate and social impacts.

## Abbreviations

CO_2_e: carbon dioxide equivalent
HuIA: human impact assessment
